# Environmental associations of *Ophidiomyces ophidiicola*, the causative agent of ophidiomycosis in snakes

**DOI:** 10.1371/journal.pone.0310954

**Published:** 2024-10-22

**Authors:** Nicholas Friedeman, Evin Carter, Bruce A. Kingsbury, Michael J. Ravesi, Jillian M. Josimovich, Monica Matthews, Mark A. Jordan

**Affiliations:** 1 Department of Biological Sciences, Purdue University-Fort Wayne, Fort Wayne, Indiana, United States of America; 2 Environmental Sciences Division, Oak Ridge National Laboratory, Oak Ridge, Tennessee, United States of America; 3 Wildlife Division, Connecticut Department of Energy and Environmental Protection, Hartford, Connecticut, United States of America; 4 Avon Park Air Force Range, U.S. Fish and Wildlife Service, Avon Park, Florida, United States of America; 5 Santa Barbata County Fire Safe Council, Santa Barbara, California, United States of America; BOKU: Universitat fur Bodenkultur Wien, AUSTRIA

## Abstract

Emerging pathogenic fungi have become a topic of conservation concern due to declines observed in several host taxa. One emerging fungal pathogen, *Ophidiomyces ophidiicola*, is well documented as the causative agent of ophidiomycosis, otherwise known as snake fungal disease (SFD). *O*. *ophidiicola* has been found to cause disease in a variety of snake species across the United States, including the eastern massasauga (*Sistrurus catenatus*), a federally threatened rattlesnake species. Most work to date has involved detecting *O*. *ophidiicola* for diagnosis of infection through direct sampling of snakes, and attempts to detect *O*. *ophidiicola* in the abiotic environment to better understand its distribution, seasonality, and habitat associations are lacking. We collected topsoil and groundwater samples from four macrohabitat types across multiple seasons in northern Michigan at a site where *Ophidiomyces* infection has been confirmed in eastern massasauga. Using a quantitative PCR (qPCR) assay developed for *O*. *ophidiicola*, we detected *Ophidiomyces* DNA in topsoil but observed minimal to no detection in groundwater samples. Detection frequency did not differ between habitats, but samples grouped seasonally showed higher detection during mid-summer. We found no relationships of detection with hypothesized environmental correlates such as soil pH, temperature, or moisture content. Furthermore, the distribution of *Ophidiomyces* positive samples across the site was not linked to estimated space use of massasaugas. Our data suggests that season has some effect on the presence of *Ophidiomyces*. Differences in presence between habitats may exist but are likely more dependent on the time of sampling and currently uninvestigated soil or biotic parameters. These findings build on our understanding of *Ophidiomyces* ecology and epidemiology to help inform where and when snakes may be exposed to the fungus in the environment.

## Introduction

Emerging infectious disease is a recognized threat to global biodiversity [1–3]. Among such diseases, fungal pathogens have come to the forefront having appeared in several forms, most notably amphibian chytridiomycosis (*Batrachochytrium dendrobatidis*) and white nose syndrome (*Pseudogymnoascus destructans*) in hibernating bats. Both diseases and their associated pathogens have caused population declines in susceptible species and displayed the ability to exist at some capacity in the environment within abiotic reservoirs [4, 5]. Persistence in abiotic reservoirs is a factor that aids in pathogen dispersal across the landscape and creates challenges for developing successful management plans to mitigate the negative impacts of disease outbreaks.

More recently, a new disease known as ophidiomycosis, has emerged. Commonly known as snake fungal disease (SFD), it has been observed in dozens of species across the eastern, midwestern, and southwestern United States (and Puerto Rico), as well as Germany, the United Kingdom, and Australia [6–15]. Recently, SFD was documented for the first time in California [16], and new cases will likely emerge in undiscovered areas in the future as monitoring continues. SFD most often appears as dermal mycosis in the form of lesions on various locations around the body. If disease progresses, lesions can develop into localized regions of necrotic tissue [17] and spread to internal structures such as the lungs and bone [13, 18]. SFD was first observed and confirmed in free ranging snakes in 2008, within a population of eastern massasauga (*Sistrurus catenatus*) in Illinois [6]. However, there were outbreaks of fungal infection consistent with SFD in both pygmy rattlesnakes (*Sistrurus miliarius*) and timber rattlesnakes (*Crotalus horridus*) in the early 2000s and 2006, respectively [10, 19]. Furthermore, examination of museum specimens has documented ophidiomycosis in snakes up to 55 years prior to the previously reported emergence [20]. Recent genetic analysis and molecular dating suggest *O*. *ophidiicola* has been introduced to North America multiple times from an unknown source population, some of those introductions occurring within the last couple hundred years [21]. Such findings add to the story of *O*. *ophidiicola* in North America and suggests introduction and more long-term existence of the pathogen than is suggested field and historical observation.

Since the first documented observation of SFD, *Ophidiomyces ophidiicola* has been identified as the causative agent through phylogenetic analysis and experimental infection of snakes [17, 22]. *O*. *ophidiicola* exhibits a wide array of metabolic activity (lipases, gelatinases, keratinases, etc.), has the ability to grow on the dead tissue of several taxa, and uses a variety of substrates [23]. Additionally, *Ophidiomyces* can grow under a wide range of temperature (7°C to 35°C), pH (5–11), and moisture conditions [23]. This evidence has led to the hypothesis that it can live as a saprotroph in the environment. Recently, *O*. *ophidiicola* has been detected within topsoil and sediment samples from snake hibernacula, indicating that it is present to some extent within the soil environment [24–26]. Furthermore, growth assays indicated that *Ophidiomyces* has the potential to grow in sterile soils but not in soils where the native microbiome was left intact [25]. Although *Ophidiomyces* may not display the competitive capacity needed to exist as a ubiquitous saprobe within soil [25], soil may serve as a reservoir of fungal spores [27]. Continued investigation into soil and other potential environmental reservoirs is warranted.

To date, most literature on *O*. *ophidiicola* comes from direct surveying and swabbing of snakes. There has been little work aimed at environmental surveying and detection. Filling in the information gaps on *Ophidiomyces* ecology and the potential for environmental reservoirs is critical to enhance our understanding of this fungal pathogen. For that reason, we sought to investigate whether *Ophidiomyces* was present in topsoil and groundwater independent of snakes at a site where SFD is known to occur [7, 9] and ascertain if certain environmental parameters were correlated with its presence. Additionally, we aimed to assess spatial or temporal differences in fungal presence within the site and determine if the observed distribution of *O*. *ophidiicola* is associated with estimated massasauga space use (a susceptible species occupying the sampling site). Such information can help us understand how snakes are exposed to the pathogen, giving information for managers actively surveying for *Ophidiomyces*.

## Methods

### Ethics statement

This work constitutes an observational and field study and was carried out on a portion of publicly accessible land belonging to the Michigan National Guard (more specific location details will be withheld to protect sensitive species location information). In this study a subset of data came from previous research at the same site where eastern massasauga rattlesnakes, federally threatened and listed as a species of special concern in Michigan, were captured and radio-tracked to collect spatial data throughout their active seasons. This represents data that was collected in the past, which is being applied in this manuscript. No permits were required for the current work, which consisted of the main data set consisting of environmental sampling of soil and groundwater to detect fungal presence. This is because environmental sampling occurred on publicly accessible land and was separate from any work involving protected species. In addition to permitting, all sampling and previous snake radio-tracking was carried out and approved with/by the environmental office of the national guard facility.

### Survey design

Sampling was conducted in northern Michigan at a site that is home to several species protected at the state and federal level. The area of interest was approximately 10.5 km^2^ and was developed around known eastern massasauga habitat. We sampled topsoil (July ‐ November of 2020 and April ‐ May of 2021) and groundwater (July ‐ November of 2020 and April ‐ June of 2021) on a temporal basis, splitting sampling across three seasons: spring (April-May), summer (July ‐ August), and fall (September ‐ November). Sampling was also conducted spatially following a three-level design involving primary units, secondary units, and technical replicate observations. Primary units consisted of macrohabitats of interest delineated through supervised image classification on land cover data (United States Geological Survey), as well as thorough inspection of aerial imagery (ArcGIS version 10.5, Esri) and on-site proofing. Four macrohabitats were chosen for sampling: forest (closed canopy coniferous and deciduous), shrub-scrub open (areas with <30% canopy cover dominated by woody shrubbery), burn (area previously burned in 2010), and cuts (areas of pine and aspen clear cuts). These macrohabitat types were chosen as the primary units of sampling to ensure we captured distinct habitats that may have different capacities to harbor *O*. *ophidiicola* but prevent spreading the sample size and losing statistical power. The sampling of groundwater was focused on an estimated massasauga hibernacula zone [28]. This is due to the fact that massasauga at the site will often be submerged to some extent in ground water while within hibernacula [29]. Therefore determining *O*. *ophidiicola* presence in ground water only has direct, transmission level significance within areas their hibernacula are present. In addition, ground water sampling was more demanding and time consuming. Therefore, having a single area to focus ground water sampling enabled us to capture more detail. Secondary sample units consisted of randomly chosen topsoil and groundwater sampling locations within each primary unit. The third level was a series of three technical replicates on each sample using the real time PCR.

### Sample collection

Topsoil samples were collected during multiple trips made to the field within each sampling season. Samples consisted of ∼40g of soil that were dug from a circular area 10cm in diameter by 10cm in depth. Collection was completed using sterile spatulas, and collected topsoil was placed in sterile Fisherbrand® 4 oz. specimen containers. All samples were kept on ice for short term storage, typically 1–3 days prior to processing [30]. If long term storage of one week or longer was required, samples were kept at -80°C. We collected 215 topsoil samples among the four macrohabitats. At each sampling point we measured soil pH (Soil pH), soil temperature at 10cm depth (Soil t), percent soil moisture (moisture), and percent soil organic matter (organic) for downstream occupancy analysis of hypothesized correlates of *Ophidiomyces* presence.

We collected groundwater samples within the hibernacula boundary [28] during each season through multiple trips to the site. Sampling required the use of a steel drive point well system (Solnist®). The well apparatus consisted of a 30cm piezometer and a 60cm extension pipe. Piezometers were lined with mesh (304 S.S. 50 Mesh, 0.254mm) to filter groundwater while excluding large sand and debris particles. Water depths at massasauga hibernacula from previous measurements average around ∼0.5m and range between 0–0.9m in depth, fluctuating year to year [29]. Samples were taken at a depth of ∼1m to ensure that enough water could accessed and drawn at each sampling point throughout the hibernacula sampling area [29]. Once in the ground, water was pumped using a peristaltic pump head, filling a sterile 500ml Nalgene bottle. Samples were stored in the same manner as topsoil samples. A second, paired water sample was taken after the sample was drawn to measure water temperature, dissolved oxygen (mg/L), and pH. Since wells were used between different sampling locations, all equipment that had direct contact with groundwater was soaked in a 3% sodium hypochlorite (50% bleach) solution for up to 20 minutes and rinsed with water. Decontaminated equipment was transported to the field in separate containers from used equipment.

Prior to DNA extraction, groundwater samples were filtered through a Nalgene® 250ml rapid flow filter unit with a 0.45μm nitrocellulose filter membrane to capture DNA and fungal material present in water samples. Filtration commenced immediately upon return to the lab. It took up to 5 days to filter all samples collected during a given trip. If samples were not filtered within the first day, they were stored at 4°C until they could be processed. Filtered samples were stored at -80°C until extraction.

We collected field controls for each sampling event. Positive controls consisted of field collected soil and groundwater spiked with 1.0 x 10^5^ copies of plasmid containing the target gene. Field negatives consisted of DNA free sand (Sigma-Aldrich) and deionized water brought to the field and collected utilizing the same approach as regular samples. All field controls were stored under the same conditions as environmental samples.

### Extraction and *O*. *ophidiicola* detection

We utilized the Qiagen DNeasy® PowerSoil® Pro Kit for DNA extraction from both topsoil and groundwater following the manufacturer protocol. Bulk topsoil samples were homogenized and 25mg of the total sample was loaded into the extraction tubes with sterile forceps. Groundwater filters were torn with sterile forceps and both filters and accumulated sediment on the filters was placed into the extraction tubes. After completion, samples were eluted in 100μl of elution buffer and stored at -80°C. We measured DNA concentrations (ng/μl) of samples using spectrophotometry (NanoDrop^TM^, Thermo^TM^ Scientific).

All extracted samples were screened on a (insert qPCR machine) using a real-time PCR assay developed to target the internal transcribed spacer region (ITS) of *O*. *ophidiicola*, a region existing between rRNA genes [31]. Each sample plate included a standard curve, a series of DNA standards made from linearized plasmid containing the ITS region. Standards were run in triplicate and consisted of 10-fold serial dilutions in a range from 1.05 x 10^7^ to 1.05 x 10^1^ copies of the ITS insert. An internal positive control (Applied Biosystems TaqMan Exogenous Internal Positive Control) was run within all reactions to monitor PCR inhibition. To ensure reduced contamination during real-time PCR, all PCR related reagents were handled in a class 2 biological safety cabinet away from where samples were stored and processed. This allowed for easy decontamination before and after each use.

Samples and their respective replicates were assigned a binomial indicator (1 = positive, 0 = negative) of detection status. Samples were determined positive if at least one replicate amplified below the cycle threshold (CT) of 40 based on suggestions made in Bohuski et al. (2015) and applied in Campbell et al. (2021). All suspected positive samples were Sanger sequenced (MCLAB) to confirm amplification of the correct sequence from *O*. *ophidiicola*. The small product size from the assay caused direct Sanger sequencing from PCR product to be unreliable. Therefore, 5’ extensions consisting of M13 standard primers (-21F, -48R) were added to existing primers to lengthen the total size of the product. These modified primers were run in a subsequent round of PCR after using the original primer set in the first PCR reaction. The lengthened PCR products were purified from an agarose gel using a Qiagen QIAquick® Gel Extraction Kit prior to sequencing.

### Spatial and temporal comparison of detection

*O*. *ophidiicola* detection in a sample was assessed on a spatial scale by comparing positive detections among the macrohabitat types. To draw conclusions about temporal patterns in *O*. *ophidiicola* detection and presence, we also grouped samples into three seasonal categories regardless of macrohabitat: spring (April-May), summer (July ‐ August), and fall (September ‐ October). Given the binomial detection data, a binomial logistic regression (R package stats 4.2.1) was applied to determine the main and interactive effects that macrohabitat type and season had on detection at the site. The R package, emmeans 1.9.0 was applied with the Tukey method for multiple comparisons to assess where potential differences existed for main and interaction effects when significance was found.

### Occupancy modeling to determine environmental correlates

To determine *Ophidiomyces* occupancy and environmental parameters that may predict its presence, we generated Bayesian, multi-scale occupancy models utilizing the eDNAOCCUPANCY (Dorazio and Erickson 2018) package in R statistical software. We assumed a multivariate standard normal distribution prior in all models. Models were fitted using Markov Chain Monte Carlo (MCMC) algorithms, each running for a total of 11,000 iterations. Model convergence and autocorrelation was assessed with trace plots and autocorrelation functions, both of which were provided within the package framework [32]. Three probabilities based on the posterior means of derived model parameters were estimated: the occurrence probability at the site level, the conditional probability of occurrence in a sample, and the detection probability in replicates from a sample, represented as Ψ(psi), ϴ(theta), and p, respectively.

The generated model set consisted of models with measured variables thought to inform *Ophidiomyces* presence included as covariates within the models. Additionally, a null model with no covariate measures was included. Due to the high number of hypothesized variable effects, a two-step approach was applied [33–35] to reduce the number of models considered in the full set. Detection probability p(x) was modeled first with occupancy at the site and sample level held constant (Ψ(), ϴ()). Covariates investigated in modeling of detection probability were those hypothesized to affect PCR inhibition including bulk DNA concentration in ng/μl (DNA.con), percent soil organic matter (organic), and soil pH (Soil pH). After model selection, the top model for detection probability Ψ(), ϴ(), and p(x) was then run through subsequent occupancy modeling. Covariates of occupancy in a sample were hypothesized to influence presence. Soil pH, organic matter (organic) and soil moisture (moisture) all have known effects on fungal and microbial ecology within soils [23, 36–38]. Soil temperature (Soil t) is another influentinal factor to consider when looking at microbial presence in soils. We incorporated two additional covariates to capture temporally derived difference in detection: cumulative growing degree day (cGDD) three weeks prior to sampling and total precipitation in mm (weekly prcpn) one week prior to sampling. cGDD provides a possibly more useful measurment of the capacity for fungal growth compared to conventional meaures such as Julian date [39–41]. Precipitation is another useful measurement, as fungal growth and activity has been shown to increase following soil wetting events [38].

Models within the candidate set were scored based on the multiple selection criterion provided in the modeling framework using Watanabe Akaike information criterion (WAIC) and posterior predictive loss criterion (PPLC). Formal model parameters beta, alpha, and delta generated with each model represented the effect of regressors on the occurrence and detection probability estimates at each scale. Estimates of those formal parameters and derived parameters Ψ, ϴ, and p were taken from the most favored model under both criterion and used to produce the overall occupancy and detection probability estimates.

### Massasauga utilization distribution

Several studies related to massasauga habitat selection and habitat suitability have occurred at the site we sampled for *Ophidiomyces* [28, 42–44]. Consequently, there is a large telemetry data set present for massasauga at the site (2002–2018). Our interest was to utilize the existing telemetry data to estimate space use by massasaugas and use that information to assess if it is related to the distribution of positive *Ophidiomyces* detections. To accomplish this, we used telemetry data consisting of 751 snake locations from 19 snakes tracked during their activity season (May to August) from 2016–2018, a period closest to our soil surveys. Of the 19 snakes, there were ten males, five non-gravid females, and four gravid females included in analysis. All eastern massasauga within the tracking data set were tested for the presence of *O*. *ophidiicola* and docmented over the course of each tracking season.

To estimate space use of snakes, we generated dynamic Brownian Bridge movement models (dBBMMs) using Move [45] in R statistical software (R version 4.2.1) to estimate a utilization distribution (UD) for each snake. dBBMM models have been applied in several studies working with snake movement data [46–50] offering advantages over traditional space use estimators such as minimum convex polygon and kernel density estimators [48]. Animal trajectories, depending on the duration of study, often display changes in behavioral states that influence movement through a landscape. The use of dBBMMs provides advantages over use of standard BBMMs in their ability to estimate UDs for heterogeneous animal tracks [48, 51] as the motion variance (σ^2^) can vary over time based on the user’s discretion of sampling windows.

For our dBBMM model, we utilized previous knowledge on massasauga movement behavior at the site [52] in addition to recommendations made in Kranstauber et al. (2012) to inform model parameters. Tuning the model parameters based on the tracking data collected and movement behavior of the species can provide more accurate model estimates, maximizing the stability of σ^2^ while providing more biologically significant estimates to the species of interest. The eastern massasauga tracking data we used for analysis had relatively coarse temporal resolution with relocations occurring two–three times a week, making it more difficult to target finer windows of the snake trajectories for the dBBMMs. Becasue of this we selected a window size of 11 representing approximately one month of relocations and a margin of 3 (targeting shifts between directed movement and foraging). The location error was set at as the standard measuring accuracy of the GPS units used (3 m).

We generated UDs for each snake representing the minimum area in which there is a given probability that a snake was located there. Contours were added to each snake UD in the form of 95% and 50% isopleths (i.e., activity area including corridors for movement and core areas, respectively).

Once an estimate of massasauga space use for each snake at the site was developed with the dBBMM UD, we investigated the relationship between that space use and *Ophidiomyces* positive detection in topsoil. To accomplish this, a Euclidean distance approach like that used in habitat selection studies [53, 54] was applied. We calculated the distance from each negative sampling point and observed positive point to the nearest portion of each estimated massasauga UD using the Euclidean distance spatial analyst tool (Arc GIS pro-2.9.0). We then averaged the distance values to each snake UD for both positive and negative sampling points. Average distance values were assessed for both normality (Shapiro-Wilks, R package stats 4.2.1) and homogeneity of variance (Levene-test, R package car 3.1–2). Given the data distribution, the mean was used as the measure of central tendency and the standard deviation as the measure of variance. A Welch’s two sample t-test (R package stats 4.2.1) was utilized to compare the mean distance between observed positive and negative locations across all snakes. The threshold of statistical significance was set at α = 0.05 for all analyses.

## Results

### *O*. *ophidiicola* detection

Of the 215 topsoil samples collected across four macrohabitat types, 32 (14.8%) showed signs of amplification below the threshold CT value (S1 Table). All 32 positive samples were confirmed by sequence alignment to the target region (Geneious 11.1.5) and had greater than 95% sequence identity to *O*. *ophidiicola* reference data (Genbank). Only one out of 50 groundwater samples yielded a positive, so further analysis was not conducted. All field controls ran as expected with no amplification from negative controls, while all positive controls ran consistently with their corresponding environmental samples. The internal positive control (IPC) within each reaction ran consistently for all samples throughout all plates.

### Spatial and temporal differences in detection

**Fig 1:**
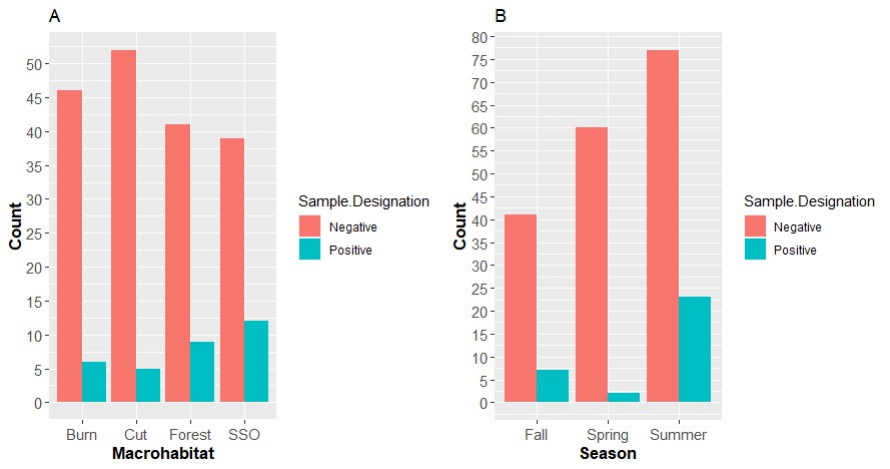
Proportion of *Ophidiomyces ophidiicola*-positive and negative topsoil samples collected from each macrohabitat type (A) and season (B) in northern Michigan during 2020–2021. Macrohabitats consisted of forested (Forest), shrub-scrub open (SSO), and two modified habitats: burned area (Burn) and clear cuts (Cut). While seasons denote samples taken during April-May (Spring), July-August (Summer), and October-November (Fall).

Detections in topsoil varied among macrohabitats, with confirmed positives occurring in 23.5% (12/51), 18% (9/50), 11.5% (6/52), 8.8% (5/57) of Shrub-scrub open, Forest, Burn, and Clear-cut habitats, respectively (Fig 1A). However, this variability lacked statistical support (S2 Table). Between seasonal groupings the summer season had the highest positive detection with 23.0% (23/100) followed by fall having 14.6% (7/48) and spring with 3.2% (2/62) (Fig 1B). No significant differences in detection were observed between both spring/fall (Coefficient = -1.634, SE = 0.827, *p* = 0.118) and summer/fall (Coefficient = -0.559, SE = 0.473, p = 0.453), but detection was significantly higher in the summer than in the spring (Coefficient = -2.193, SE 0.757, p = 0.010) (S2 Table), which had the lowest probability of detection at 3.2%. We identified a significant habitat and season interaction on positive topsoil detection with higher detection occurring in Forest and SSO habitats during the summer (Forest: Summer p = 0.036, SSO: Summer p = 0.047) relative to others. However, when post-hoc analysis was done to view differences between habitats within each season, no significant interaction was seen (S3 Table). Additional investigation into the seasonal patterns of measured soil parameters found that both soil temperature and soil pH exhibited upward shifts during the summer season in contrast to fall and spring (S1 Fig).

### Determination of environmental associations

The candidate set for modeling of covariate effects on *Ophidiomyces* detection probability consisted of eight total models (Table 1). Within the model set, the null model p() with detection probability held constant had the most support (WAIC = 69.62, PPLC = 66.35), suggesting that no covariate influenced detection. Under this model the detection probability (p) and its 95% Bayesian confidence interval (BCI) was 0.33 (BCI, 0.20, 0.46).

**Table 1:** Candidate models for probability of detection (p) of *Ophidiomyces* DNA in PCR replicates.

Model	WAIC	PPLC
**Ψ(),Θ(),p()**	**67.75**	**63.02**
Ψ(),Θ(),p(organic)	71.16	65.12
Ψ(),Θ(),p(DNA.con)	75.32	71.21
Ψ(),Θ(),p(Soil pH)	75.85	70.02
Ψ(),Θ(),p(Soil pH+organic)	77.95	70.53
Ψ(),Θ(),p(DNA.con+organic)	78.31	73.44
Ψ(),Θ(),p(Soil pH+DNA.con)	78.95	74.43
Ψ(),Θ(),p(DNA.con+organic+Soil pH)	81.78	75.95

Models ordered by WAIC criteria. Covariates tested include organic matter content (organic), DNA concentration (DNA.con), soil pH (Soil pH). Model in bold font represents top ranked model used to derive probability of detection.

**Fig 2:**
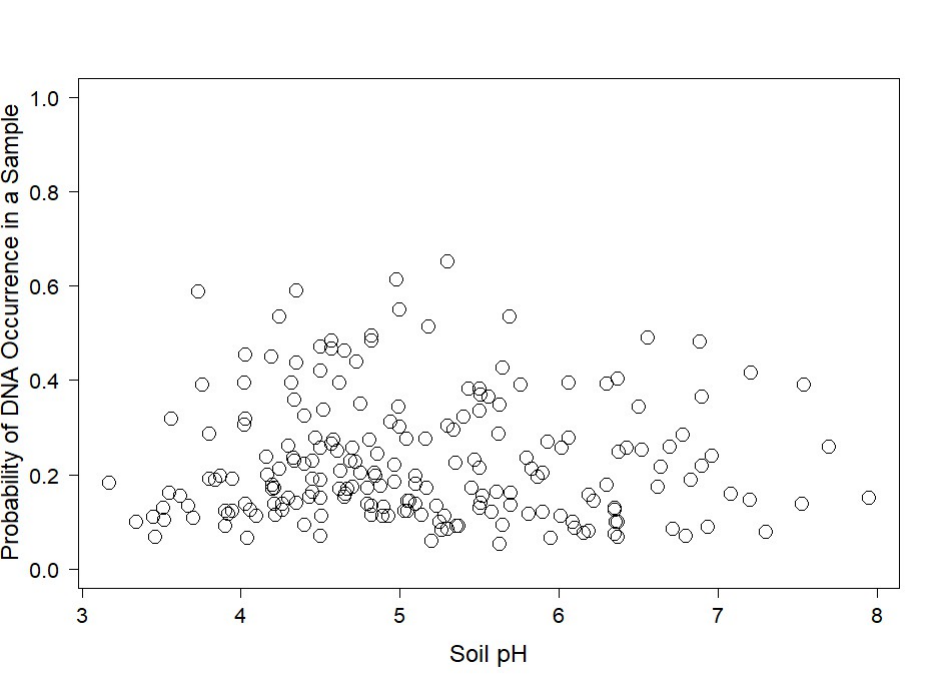
Relationship between the probability of *Ophidiomyces ophidiicola* DNA occurrence in each sample and pH of the topsoil collected in northern Michigan during 2020–2021. Each point represents the posterior mean estimate from a sample (occupancy probability Θ) at a given pH. 95% credible limits showed high variability and are not included for ease of viewing.

The occupancy model set contained 63 total models (S4 Table) and incorporated all potential effects from covariates hypothesized to inform *Ophidiomyces* presence, while using the null for detection as discussed above. The top ten models form that candidate set are displayed in Table 2. The two selection criteria applied in the modeling framework favored the null model Ψ(),Θ(),p() (Table 3) (WAIC = 67.75, PPLC = 63.02). The next best model Ψ(),Θ(Soil pH),p() included soil pH as a covariate of sample occupancy. The occupancy probability of *Ophidiomyces* at the site level (Ψ) was assumed constant and estimated as 0.83 (BCI, 0.47, 0.99), while the conditional probability of occupancy at the sample level (Θ) ranged from 0.05 to 0.59 (S5 Table). The formal model parameter alpha(Soil.pH), which refers to the effect of regressors on the probability of occupancy in a sample, indicated that occurrence of *Ophidiomyces* DNA in a sample increase with increasing soil pH (mean = 0.442, 95% BCI = 0.188, 0.728, Table 3). However, when (Θ) is plotted against soil pH there appears to be no discernible relationship (Fig 2).

**Table 2:** Candidate model set for occupancy at the sample level (Θ).

Model	WAIC	PPLC
**Ψ(),Θ(),p() (null)**	**67.75**	**63.02**
Ψ(),Θ(Soil pH),p()	67.80	63.68
Ψ(),Θ(Soil pH+weekly prcpn),p()	68.29	64.30
Ψ(),Θ(moisture),p()	68.57	64.01
Ψ(),Θ(Soil pH+cGDD),p()	68.59	64.59
Ψ(),Θ(weekly prcpn),p()	68.72	64.14
Ψ(),Θ(Soil t),p()	68.74	64.41
Ψ(),Θ(Soil pH+moisture),p()	68.86	64.92
Ψ(),Θ(Soil pH+Soil t),p()	68.92	64.81
Ψ(),Θ(Soil pH+Soil t+cGDD),p()	69.16	65.13

Models scored based on selection criteria WAIC and PPLC included within the package framework. Depicts 10 of 63 models within the candidate set. Bold font indicates the top ranked model based on each selection criteria.

**Table 3:** Summary of the formal parameters of selected MCMC models.

Bayesian Estimates of Model Parameters			
Model		Mean	95% CL
**Ψ(),Θ(),p()**	β (Intercept)	1.16	-0.057, 2.588
	α (Intercept)	-0.774	-1.060, -0.413
	δ (Intercept)	-0.448	-0.838, 0.097
**Ψ(),Θ(Soil pH),p()**	β (Intercept)	1.160	-0.078, 2.576
	α (Intercept)	-0.831	-1.139, -0.457
	α (Soil pH)	0.442	0.188, 0.728
	δ (Intercept)	-0.462	-0.834, 0.099

Beta, alpha, and delta represent the regressor effect on estimates for the site, sample, and replicate sampling levels, respectively. Model Ψ(),Θ(),p() shows estimates for the null model with no covariates. Model Ψ(),Θ(Soil pH),p() relates to the model with soil pH as a covariate of sample occupancy.

The results of the null model Ψ(),Θ(),p() (Table 3) was used to derive the final occupancy and detection probability estimates 0.83 (BCI, 0.48, 0.99) and 0.22 (BCI, 0.14, 0.34) for Ψ and Θ, respectively.

### Massasauga space use and its relation to *O*. *ophidiicola*

**Fig 3:**
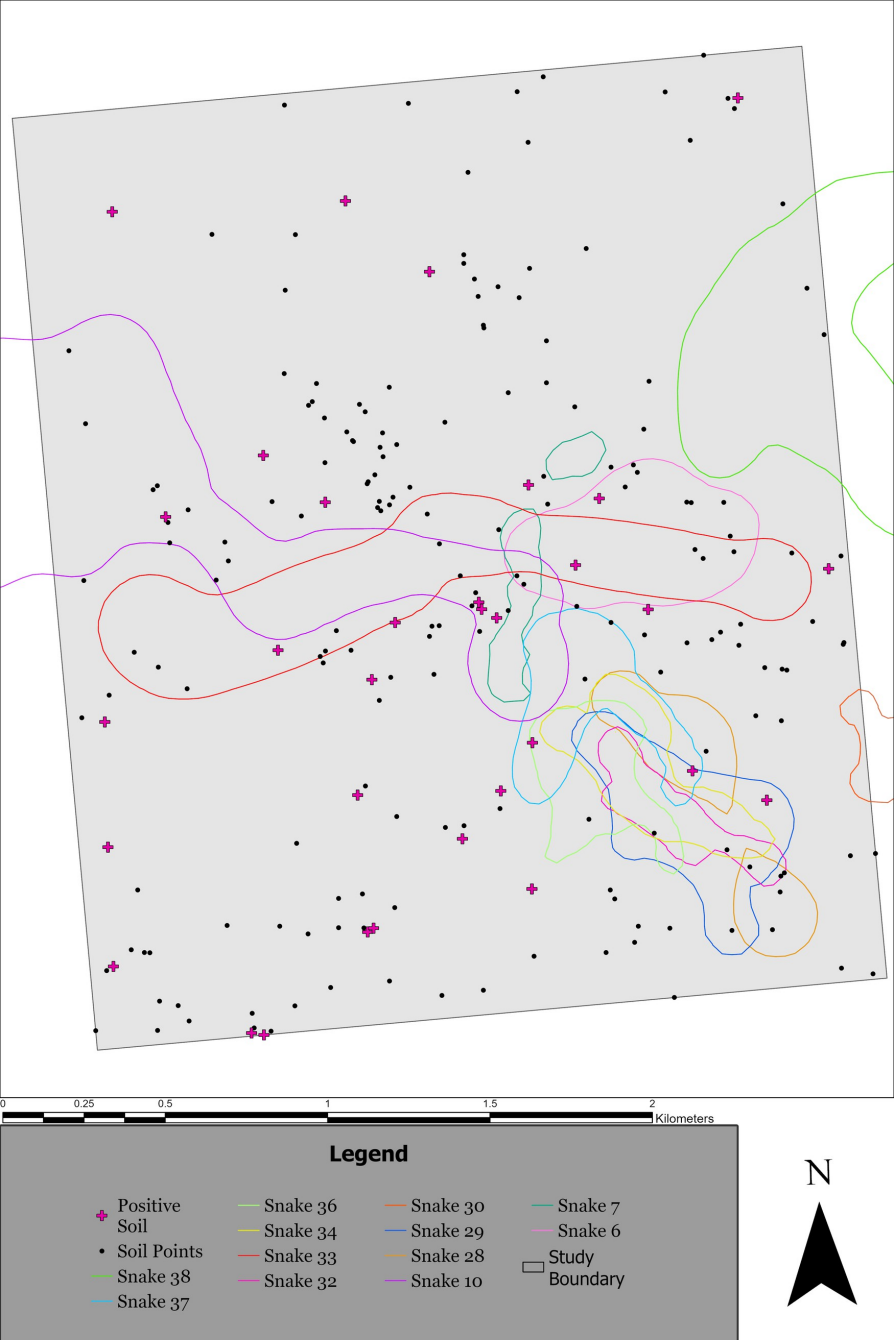
Utilization distribution (UD) for each eastern massasauga (*Sistrurus catenatus*) tracked at the study site in northern Michigan from 2016–2018. Massasuaga UDs are viewed relative to *Ophidiomyces ophidiicola*-positive locations (purple dots) and -negative locations (black dots). For viewing simplicity, only the 95% isopleths of each snake analyzed are presented here.

Massasauga activity season UD was estimated for all telemetered snakes at both 95% and 50% “core area” contours. The merged output of all individual snake UDs shows the estimated massasauga space use within and outside of the *Ophidiomyces* sampling area (Fig 3). We found that of 32 *Ophidiomyces* positive topsoil samples, 31% of them occurred within estimated 95% UD contours (10/32), while no positive topsoil samples occurred within the 50% “core area” contours (0/32).

Distance measures for the observed positive and negative points exhibited normality (Shapiro Wilk: W = 0.92, p = 0.095) and equal variance (F = 0.045, p = 0.834). *Ophidiomyces* positive topsoil samples were not found to be associated with massasauga use we estimated. There was no difference in the mean distance of *Ophidiomyces* positive samples (mean = 828.31, SD = 285.19) and random points (mean = 829.51, SD = 243.52) to the 95% snake UDs (t = -0.011, df = 21.47, p = 0.991).

## Discussion

Since the discovery of SFD and the identification of its causative agent *O*. *ophidiicola*, fewer efforts have been made to determine its ability to persist in the environment independent of snakes. Our results demonstrate that detection of *Ophidiomyces* is possible in topsoil samples within a snake population confirmed with SFD presence [9]. The revalation that *O*. *ophidiicola* can and does exist in topsoil is not entirely surprising. Other fungal pathogens such as *B*. *dendrobatidis* and *P*. *destructans* have shown the ability to grow and persist in the environment independent of a host [4, 55] add Reynolds and Kolby. In contrast to those systems however, environmental testing for *O*. *ophidiicola* has not been thoroughly explored. This study represents one of the few sucessful attempts to detect and confirm presence of *O*. *ophidiicola* in evnironnmental mediums [24, 25].

Our detection of *Ophidiomyces* in topsoil in northern Michigan, like that in the New Jesery pinelands [24, 25], idicate that is is present in the evnironment and may fuction as a potential resevoir for the pathogen. *O*. *ophidiicola* was present within 14.8% of our topsoil samples. Those positives were well distributed throughout the site. Our occurence estimate for the site level was shown to be relatively high (83%). However, both occurence in a given topsoil sample and qPCR related detection probability had lower estimates (21% and 37%, respectively). The site we utilized has had confirmed *Ophidiomyces* infection within snakes for over a decade now [9]. Such a long history with the fungal pathogen offers a potential explanation as to the wide distribution and high site occurence we observed in topsoil samples. Our sample occurance and detection probability were found to have much lower estimates. Alterations to our study protocol, such as an increased number of technical replicates, could result in improvements to said estimates. Improvements that would decrease the higher false negative rate associated with low detection probability, and help increase the utility of our occupancy modeling. Additionally, because we did not attempt to culture fungus from samples, we cannot determine if positive detection in our study is due to the presence of viable *O*. *ophidiicola* or fungal DNA. However, the presence of DNA indicates that either viable fungi were present at a point in time or that shedding of fungal DNA from an infected host occurred. For that reason, our detection results still inform *Ophidiomyces* distribution at the site.

In our effort to determine spatial and temporal differences in presence we found that macrohabitat types did not influence rates of positive detection, however the time of year had some effect with detection highest through the summer season. The upward shift in both soil temperature and soil pH at the site during the summer season brought both parameters closer to conditions considered optimal for *Ophidiomyces* as determined *in-vitro* [23]. It is possible that more optimal growth conditions during this time could explain the higher number of detections.

The lack of detection within groundwater samples was somewhat perplexing as massasaguas and other snakes overwinter in groundwater and are often observed to have high levels of *Ophidiomyces* following spring emergence [7, 56, 57]. We observed only one positive groundwater sample through screening via real-time PCR and all sequencing attempts made to confirm *O*. *ophidiicola* presence in the sample failed. A possible explanation for the lack of detection in groundwater could be that sampling at ∼1m depth is outside the distribution of the fungus in this microhabitat. *Ophidiomyces*, if present in groundwater, could occur at depths closer to the surface where contact with snakes actively shedding fungal DNA could be more likely [29]. Alternatively, *Ophidiomyces* may have little to no presence in groundwater. Fungal DNA detection from water samples has been achieved for *Batrachochytrium dendrobatidis* [4], a fungal pathogen that displays an aquatic life stage. In contrast, *Ophidiomyces* exhibits filamentous growth [22, 23] and does not have a known aquatic life stage. Previously published attempts to detect *Ophidiomyces* in the water of crayfish burrows also found no detection [58]. More thorough sampling of groundwater and soils at different depths, along with laboratory tests of potential growth and spore persistence in water media, are needed to test these possibilities.

Through our modeling, none of the parameters we included in analysis were very useful for predicting *O*. *ophidiicola* presence within our topsoil samples. We found no strong correlations between occupancy and any environmental parameter. The null model was favored within our candidate set, and additional investigation of closely ranked models with included covariates such as Ψ(),Θ(Soil pH),p(), failed to produce any truly meaningful correlations. Our inability to dicern any relationship between *O*. *ophidiicola* presence and environmetnal parameters is interesting given that the suite of soil and temporal parameters we investigated have been shown to affect general fungal activity and growth in soils [36–38]. Similar research has also found no link between abiotic soil parameters and *Ophidiomyces* detection or growth [25]. *Ophidiomyces* does exhibit a broad capacity for growth *in vitro* [23], which may explain the absence of strong relationships in these variables. Campbell et al. (2021) recently found evidence for the importance of biotic variables in determining presence of *Ophidiomyces* in soil mediums. It was noted that *Ophidiomyces* growth was inhibited in soils with intact microbial communities. Microbial communities in soil are often quite complex, and there is competition among the species present. Such competition tends to result in a reduction in available nutrients and the general suppression of growth in certain species [59], as well as specific pathogen suppression [60]. Given its inability to compete and grow in colonized soils, *Ophidiomyces* may be a pathogen better adapted to snake integument rather than soil environments.

Our ability to detect *Ophidiomyces* in topsoil throughout the site, in addition to high prevalence within the soils of snake hibernacula elsewhere [24, 25], implicate soil as the most likely source of environmental infection for snakes. Positive detection we observed appeared randomly wide-spread across the site, a location where ophidiomycosis has been confirmed in snakes for near a decade [7, 9, 57]. Its wide site distribution and likely presence in the soil of snake hibernacula provide eivdence that exposure at our site and others likely occurs in hibernacula [7, 56, 57], but also potentially through contact with contaminated topsoil during their active season, independent of hibernacula. The apparent inability of *Ophidiomyces* to compete in more complex soil microbial communities [25] mean it may be challenging for conidia (spores) or hyphae that exist in a given portion of soil to grow and spread. While this does not preclude soil from acting as a source of infection for snakes, it could affect how is able to gow and spread in the colonized soil environments.

An alternative mode of *Ophidiomyces* dispersal across the study site could be through activity and movement of infected individuals: snakes acting as primary reservoirs, shedding fungal DNA or viable conidia. If so, it is expected that fungal presence would be associated with snake activity and behavior. This can be seen to some extent within snake hibernacula, as fungal detection in hibernaculum soils has yielded higher rates of positive *Ophidiomyces* detection [25]. That finding paired with observed dermal lesions on emerging snakes [56, 57] indicates one potential link between fungal presence and snake activity. However a more complete picture on the extent to which the snakes themselves may influence disease dynamics is not well understood, especially during their active seasons. To investigate this, we sought to find a realtionship between the spatial activity of eastern massasauga and *Ophidiomyces*-positive detection, given the observation of ophidiomycosis in eastern massasauga at the site. We found no association between our positive locations and massasauga space use. Of the snakes included in analysis two tested potitive for *O*. *ophidiicola*. One individual displayed symptoms of ophidiomycosis and tested positive, the other tested positive but did not display visibile symptoms. No spatial relationship existed between those postive individuals and our observed *O*. *ophidiicola* distribution., Eastern massasaugas still likely contribute to the spread of *Ophidiomyces* to some degree, through shedding of condia, hyphal fragments, or contaminated skin into the environment during ecdysis [61] or other activities. *Ophidiomyces* has the ability to infect and cause disease in a variety of snake species [10, 13–15, 57, 62, 63]. Additional species at the study site that were not surveyed include *Thamnophis sirtalis*, *T*. *sauritus*, *Lampropeltis triangulum*, *Opheodrys vernalis*, *Storeria occipitomacculata*, and *S*. *dekayi*. Each of these species may play its own role in the observed *O*. *ophidiicola* distribution.

Our inability to determine the role of eastern massasaugas distribution on *Ophidiomyces* could be due to several factors. Our snake activity data was collected two to three years prior to our topsoil survey. These older data, while still informative, could be missing shifts in snake UD that may have occurred more recently. Additionally, using UD-based estimates only provides information on space use for a subset of radio-tracked individuals within the greater population. There are more snakes at the site than we included in analysis, potentially biasing our visualization of massasauga space use at the site. Incorperating more individuals could refine our understanding of massasauga space use at the site and generate a more complete picture of where snakes may encounter *Ophidiomyces*. It is also important to emphasize again that *Ophidiomyces* can infect a variety of snake species [10, 13–15, 57, 62, 63]. It is possible that other snake species occupying the site, in addition to other animal and human movements, contributed to the distribution of *Ophidiomyces* we observed.

## Conclusions

With this study, we build upon the existing knowledge of *O*. *ophidiicola* with focus on the role environmental mediums may have in harboring the fungus and influencing disease dynamics. Our results indicate that *O*. *ophidiicola* is present within topsoil at the study site, providing further evidence that soils can act as a reservoir and potential means of transmission to snakes. Overall occupancy at the site was estimated to be relatively high, and positive samples were randomly spread throughout the site and not significantly associated with specific macrohabitat types nor the most recent estimates of massasauga space use. Additionally, we did not recover any meaningful correlations between abiotic environmental parameters and *Ophidiomyces* presence and detection probability within a sample. We observed that detection exhibited a seasonal trend with detection lowest during the cool spring and highest during the warmest point of the year. Such a trend could be caused by seasonal changes in growth of *Ophidiomyces* or via shedding by infected snakes as they move about their activity ranges during the summer.

We have developed a protocol for future environmental surveying for *Ophidiomyces*. If future attempts are made to better understand *O*. *ophidiicola* associations within soil reservoirs, we recommend that biotic variables [25] be included in the study design. Additional work aimed at improving our understanding of the environmental shedding of the fungus by infected snakes will not only inform how and to what degree infected snakes shed fungi into the environment but also if they can acquire infection from such sources.

## Supporting information

S1 Fig Seasonal changes in two investigated soil parameters. (Above) seasonal shift in the mean soil temperature at four (black) and 10 (red) inch depth measured. (Below) seasonal shift in mean soil pH. Data taken from Michigan State University Enviroweather soil conditions data from July 2020 to May 2021.
(TIF)

S1 Table List of all positive topsoil samples. Topsoil sample designation, the macrohabitat it was collected in, the number or positive replicates from a triplicate reaction, and the mean Ct value for the given sample.
(CSV)

S2 Table Binomial logistic regression output of main effects (habitat and season) on positive *O*. *ophidiicola* detection in a soil sample. (Above) regression output from main effects. (Below) post-hoc analysis below delineating where differences occur if they exist. Significant terms bolded.
(CSV)

S3 Table Binomial logistic regression output for interactive effect of habitat and season on *O*. *ophidiicola* detection in a soil sample. (Left) regression output from interactive effects. (Right) post-hoc analysis delineating where differences occur if they exist. Significant terms bolded.
(CSV)

S4 Table Complete candidate model set for occupancy at the sample level (Θ). (CSV)

S5 Table Derived probability estimates for the second-best model Ψ()Θ(Soil pH)p(). Probability estimates at the site (Ψ), sample (Θ), detection (p) levels for the second-best model Ψ()Θ(Soil pH)p() are shown as the posterior means with Bayesian confidence intervals (BCI).
(CSV)
