# Supplementary figures and images for: Environmental associations of Ophidiomyces ophidiicola, the causative agent of ophidiomycosis in snakes
Source: PLoS One. 2024 Oct 22;19(10):e0310954. doi: 10.1371/journal.pone.0310954 (PMC11495611; doi:10.1371/journal.pone.0310954)

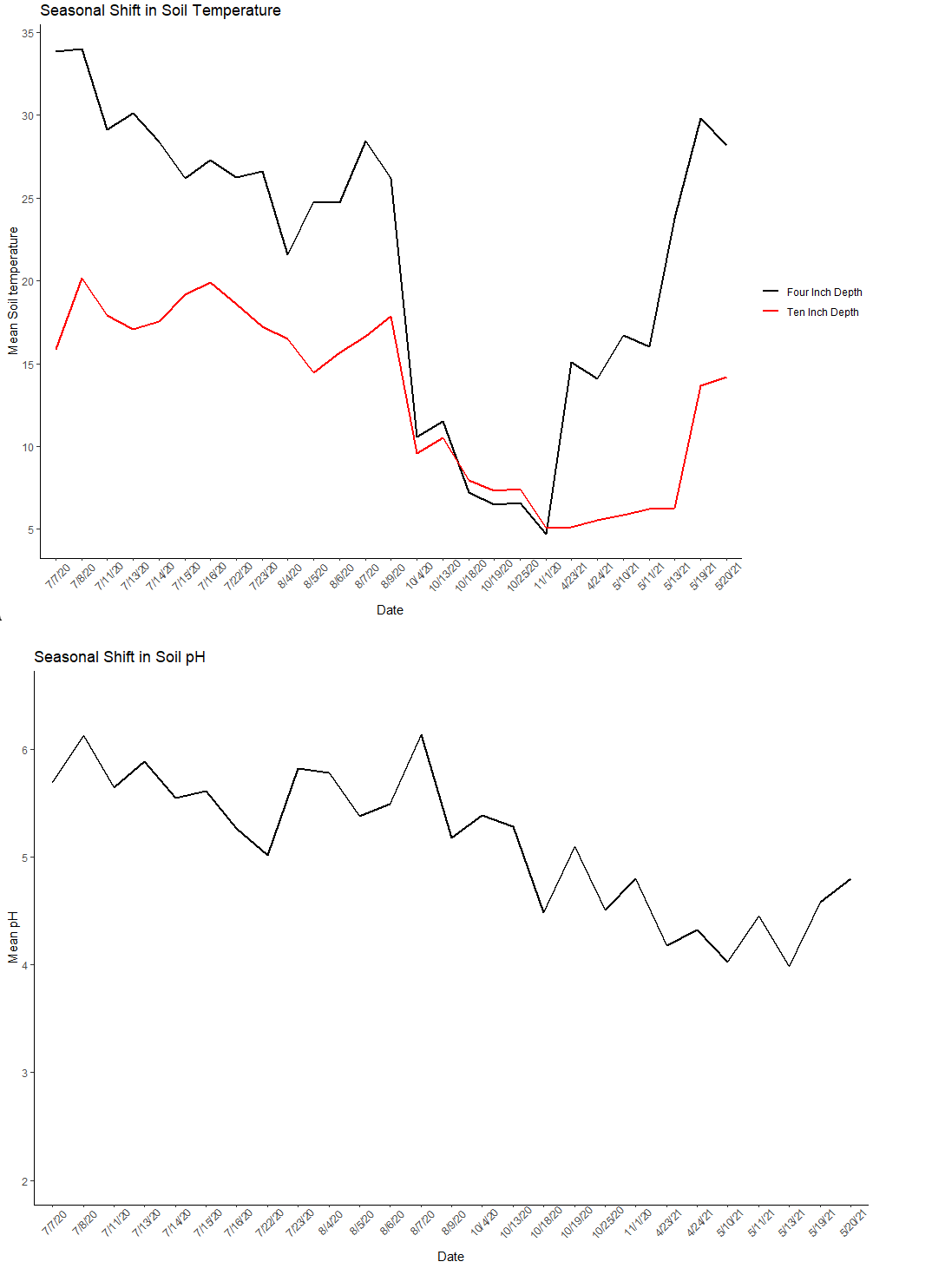

Supplement: S1 Fig — (Above) seasonal shift in the mean soil temperature at four (black) and 10 (red) inch depth measured. (Below) seasonal shift in mean soil pH. Data taken from Michigan State University Enviroweather soil conditions data from July 2020 to May 2021. (TIF) [file pone.0310954.s001.tif]
